# Pyorrhœa Alveolaris

**Published:** 1896-11

**Authors:** Eugene S. Talbot

**Affiliations:** Chicago, Ill.


### PYORRHŒA ALVEOLARIS.

BY DR. EUGENE S. TALBOT, CHICAGO, ILL.

THE October (1896) INTERNATIONAL DENTAL JOURNAL contains (Notes and Comments) the following:

> "PYORRHŒA ALVEOLARIS.—In listening to Dr. Talbot's paper upon pyorrhœa alveolaris, read before the Academy of Stomatology of Philadelphia, we noted his remark that a general invasion of this disease—that is, attacking all the teeth—'never occurs.' This is an error, as such cases frequently occur, and, no doubt, are observed by many practitioners. The writer has treated more than one case in his own practice, where every tooth was affected, and has met several in the college clinics."

Substantially the same views are expressed in an editorial in the April (1896) *Dental Cosmos* by Dr. Edward C. Kirk:

> "The statement that a general invasion of the disease, including all of the teeth, 'never occurs' is an error of fact, as such cases frequently occur, and have probably been observed by most practitioners. As a matter of fact, such a case is reported by Dr. William H. Trueman in the discusion of Dr. Talbot's paper in this issue."

These are criticisms of the following views expressed as to the uric acid theory of pyorrhœa:

> "If this theory, moreover, were correct, the deposits, which are in the capillaries in a liquid state, surrounding the teeth, would attack all the teeth at the same time, and deposit equally, or nearly so, around the root of each tooth, which never occurs."

Neither seems to understand the point aimed at. The mere reference to the deposits in the capillaries in a liquid state was sufficient to show that the incipient stage of the disease was meant, and not the stage inferred by Dr. Kirk in quoting the case cited by Dr. William H. Trueman in the discussion of my paper. I have treated many cases of the type mentioned by Dr. Kirk and the "Comment," and so has every other dentist. But this is not the point aimed at. If pyorrhœa were due to uric acid or gout, deposits would take place in the tissues about the teeth just as they do in and about the joints, and all the teeth would become affected at or nearly the same time, which is never the case.

It would take years to produce such a condition as cited in the "Comment" and the case referred to by Dr. William H. Trueman from the incipiency of simple inflammation.

Pyorrhœa is not a spontaneous disease in which all the teeth become involved at one time, but is the result of simple inflammation of the gums about one or more teeth, which become chronic with an invasion of the peridental membrane that may in time extend to all the teeth, or may be confined to one or two teeth, the deposit being the result of such chronic inflammation.

